# Predictive relationship between COVID-19 anxiety and psychological distress in adolescents during the COVID-19 pandemic

**DOI:** 10.3389/fpsyg.2023.1095892

**Published:** 2024-09-19

**Authors:** Jennifer McMahon, Katherine Dowling, Elaine Gallagher, Alanna Donnellan, Sharon Houghton, Megan Ryan, Cliodhnad O’Connor, Eibhlín Walsh

**Affiliations:** ^1^The School, Child & Youth Mental Health & Wellbeing Research Lab, Department of Psychology, University of Limerick, Limerick, Ireland; ^2^Centre for Social Issues Research, Department of Psychology, University of Limerick, Limerick, Ireland; ^3^Health Research Institute, University of Limerick, Limerick, Ireland; ^4^National Suicide Research Foundation, Cork, Ireland

**Keywords:** COVID-19, adolescents, psychological distress, pandemic anxiety, parental closeness

## Abstract

COVID-19 was a novel stressor that gave rise to pandemic related anxiety and increased the risk of mental health issues, particularly in youth. It is important to understand how such events contribute to psychological distress in young people to adequately intervene in the aftermath and to plan for future similar events. Using cross-sectional data from the CoSPACE Ireland study dataset this paper reports on the predictive relationship between COVID-19 anxiety and psychological distress for Irish adolescents (*N* = 314, *M* = 14.05, *SD* = 2.7, 11–18 years), while controlling for other influencing factors across multiple levels of a bioecological systems approach. Covariates were age, gender, ethnicity, social economic status, Peer Support, School Support and Parent–Child Closeness. Findings indicate that COVID-19 anxiety was a significant predictor of adolescents’ psychological distress. Specifically, Consequence Anxiety (worries about the indirect consequences of COVID-19) was found to be a predictor of adolescents’ psychological distress rather than Disease Anxiety (worries about the COVID-19 virus itself). Individual factors (e.g., age, ethnicity, special educational needs) and microsystem factors (e.g., parent child closeness, peer support) were also found to impact on adolescents’ levels of psychological distress. A significant moderation analysis revealed that greater parent–child closeness reduced the strength of the positive association between Consequence Anxiety and psychological distress. These findings suggest that strategies to alleviate adolescents’ psychological distress during pandemics should focus on reducing pandemic-related anxiety, specifically Consequence Anxiety. A multisystemic approach is also recommended to reduce the negative mental health impacts of the pandemic on adolescents.

## Introduction

The COVID-19 pandemic spread across the globe at an unprecedented rate and has affected the lives of millions of people worldwide (Guo et al., 2021). Negative consequences of the pandemic span further than those related to the virus itself (e.g., contracting the virus, serious illness, death etc.) and can also relate to growing concerns regarding the impact of the pandemic on populations’ mental health (Talevi et al., 2020; Nochaiwong et al., 2021; Santomauro et al., 2021). While fears around the virus itself may cause distress for individuals, additional factors such as government policies and restrictions can also negatively impact the mental health of individuals (Ghebreyesus, 2020). For example, evidence on the impacts of isolation, social distancing, and quarantine from previous pandemics (e.g., SARS, Ebola) as well as the COVID-19 pandemic has shown significant negative and long-lasting psychological effects (Brooks et al., 2020; Henssler et al., 2020).

Emerging evidence indicates that adolescents are at particular risk for developing poor mental health outcomes from the pandemic (Hossain et al., 2022; Mansfield et al., 2022; Theberath et al., 2022). According to Zhou (2020), adolescents are more susceptible than adults to the psychological effects of the pandemic because they possess underdeveloped cognitive and emotional regulation systems. Indeed, a systematic review by Nearchou et al. (2020) found that the COVID-19 pandemic has had an impact on youth mental health and de Figueiredo et al. (2021) warn that the abrupt changes in daily routines, such as school closures, lack of a social life, and lack of outdoor activities, negatively affect the mental health of adolescents both in the short and long-term. The potentially long-term consequences are particularly worrying because the biopsychosocial stressors experienced during the pandemic can negatively impact upon adolescents’ neurological development, making them more susceptible to developing psychiatric disorders in adulthood (Orben et al., 2020; de Figueiredo et al., 2021). Developing an understanding of the mental health impact of the COVID-19 pandemic and related restrictions on adolescents is therefore important to inform interventions that offset possible harm.

Existing empirical research suggests that individual factors can impact on adolescents’ likelihood of experiencing adverse mental health impacts from the pandemic. Studies have identified multiple risk and protective factors that influence the degree of psychological distress experienced by adolescents during the pandemic. For example, in a sample of Chinese adolescents, Zhou et al. (2020) found depressive and anxiety symptoms were more prevalent among female and older adolescent populations. This study also found adolescents’ awareness surrounding COVID-19 was a protective factor against these symptoms (Zhou et al., 2020). Zhou et al. (2020) study was replicated with over 1 million Chinese school-aged children and adolescents and found that in addition to age, gender and COVID-19 knowledge, engagement in physical activity and following of COVID-19 recommendations were also found to be protective factors against psychological distress (Qin et al., 2021).

Additional individual factors including Special Educational Needs (SEN), Socio-Economic Status (SES) and ethnicity can increase adolescents’ risk of experiencing higher levels of psychological distress during the pandemic. Existing evidence has indicated, for example, that adolescents with Attention Deficit Hyperactivity Disorder (ADHD) present with known risk factors (e.g., social isolation, motivation problems) for poorer mental health outcomes during the pandemic (Sibley et al., 2021). Furthermore, loss of employment and financial burden during the pandemic is more likely to impact those families of low SES and these additional stressors are likely to have negative consequences on the mental health of adolescents from these families (Darmody et al., 2020). Adolescents from minority ethnic and migrant groups may also be more likely to experience psychological distress during the pandemic due to the poverty, overcrowded conditions, stigma and discrimination they are often subjected to (Darmody et al., 2020; You et al., 2020).

In addition to individual factors, research has also pointed towards broader factors (e.g., presence of close relationships) as protecting against psychological distress in adolescents during the COVID-19 pandemic. For example, a longitudinal study which compared adolescents’ mental health prior to and during the pandemic found that while adolescents’ mental health had declined during the pandemic, higher levels of social connection predicted lower levels of anxiety, and depression and higher levels of life satisfaction (Magson et al., 2021). Conversely, adverse mental health consequences may be associated with a lack of social connectedness and subsequent feelings of loneliness in adolescents during the COVID-19 pandemic, as evidenced by a rapid systematic review (Loades et al., 2020). It is clear then that the determinants of adolescents’ psychological distress during the COVID-19 pandemic are complex and widely ranging, including both individual and broader societal factors.

One key aspect of adolescents’ psychological distress during the pandemic that has been largely neglected in the literature, relates to COVID-19 anxiety. However, a study by Lee et al. (2020) set out to examine the extent to which COVID-19 anxiety, uniquely predicts indicators of psychological distress experienced during the COVID-19 pandemic (i.e., depression, generalized anxiety and death anxiety) in an adult sample. Within this study, COVID-19 anxiety was found to be a unique predictor of psychological distress indicators during the pandemic. In another adult study, COVID-19 related anxiety was highest in those at greatest risk of mortality, i.e., over 65 s. (Hyland et al., 2020). These findings equip both researchers and healthcare professionals with the knowledge that pandemic related anxiety is a key risk factor for mental health issues in adults during the pandemic, which in turn has important implications for interventions during and following on from the COVID-19 pandemic. Given the unique developmental stage of adolescence extending our understanding of pandemic-related anxiety to the adolescent context is an important next step in identifying and treating young people at risk of developing mental health problems during a pandemic. Studies on COVID-19 anxiety tend to focus specifically on anxiety related to being infected by the disease itself and do not observe additional aspects of COVID-19 anxiety. McElroy et al. (2020) recognised that COVID-19 anxiety is multidimensional, differentiating between anxiety related to the COVID-19 virus itself (i.e., disease anxiety) and anxiety related to the associated consequences of the pandemic (i.e., consequence anxiety). Both disease anxiety and consequence anxiety can impact on the health and wellbeing of populations; however, some individuals may be more likely to experience one over the other depending on specific characteristics. For example, most COVID-19 hospitalizations and deaths affected older populations and those with underlying health conditions (Jordan et al., 2020), therefore it is not surprising that disease anxiety might be more prevalent with these groups. Similarly, McElroy et al. (2020), found that those with underlying health conditions were more likely to be concerned about the disease itself. In contrast older adolescents and those with lower income were more likely to be concerned about the long-term consequences of the pandemic (McElroy et al., 2020). Given that young people and those with less financial stability may be more likely to feel the negative consequences of the pandemic due to disrupted education, loss of employment, lack of social contact and greater uncertainty about the future (Ahmed et al., 2020) pandemic consequence anxiety is likely to be particularly salient in these populations.

To the best of our knowledge, no studies examining the degree to which aspects of COVID-19 anxiety uniquely predicts psychological distress in adolescents during the pandemic have been conducted thus far, either within the Irish context or further afield. However existing research has linked COVID-19 anxiety to a range of negative psychological and somatic outcomes in adults (Shevlin et al., 2020; Savolainen et al., 2021). To scaffold the exploration of the effect of COVID-19 anxiety in adolescents we draw on Ecological Systems Theory (Bronfenbrenner, 1979). Ecological Systems Theory (Bronfenbrenner, 1979) views human development in the context of their immediate and wider social environment (Fearnley, 2020). The theoretical framework provides a useful lens for understanding the many factors that impact on the developmental outcomes of children and adolescents. According to Bronfenbrenner (1979), human development is complex and is influenced by multiple interrelated systems within the ‘ecological environment’ namely, the microsystem (i.e., immediate environment), mesosystem (i.e., interactions of microsystems such as between home and school), exosystem (i.e., social structures) and macrosystem (i.e., wider society and culture) (Bronfenbrenner, 1979; Ashiabi and O’Neal, 2015). In this study, several microsystem factors will be examined as these have been shown to be strong protective factors against poor psychological outcomes for young people. For example, evidence shows that young people with strong supportive relationships with parents significantly impacts young people’s wellbeing and life satisfaction, while also mitigating against poor outcomes such as internalising difficulties and low mood (Oberle et al., 2011; Patrick et al., 2020; Hartas, 2021; Smyth and Nolan, 2022). While family relationships are important for young people, as they get older adolescents begin to rely on additional support from their peers and school as well (Viner et al., 2012). Friendships and peer support have also been shown to promote positive wellbeing in young people and act as a buffer or protective factor against poor psychological outcomes such as stress and anxiety (Beeble et al., 2009; Butler et al., 2022). Furthermore, several studies have found that positive supportive relationships with teachers can positively impact on students’ wellbeing and psychological outcomes, and this is particularly true for young people who experience adversity and have a lack of supportive relationships at home (Heard-Garris et al., 2018; Hughes et al., 2018).

Previous research (Guo et al., 2021) has successfully applied Ecological Systems Theory to explain the psychological distress of adults during the pandemic. Drawing on Bronfenbrenner’s theory we explore the effect of COVID-19 anxiety on young people’s psychological distress, while controlling for risk and protective factors at different levels (i.e., individual and microsystem).

### The present study

The specific aim of this paper is to assess the predictive relationship between COVID-19 anxiety and psychological distress in a sample of Irish adolescents. This study took place between April –June 2020, a time when COVID-19 restrictions were in place. Most intense. In the First Wave (February to August 2020) cases of COVID-19 increased significantly, from 8,089 in early April to 24,990 (+16,901, 523 per 100,000 of the population) in late May (Lima, 2021). At this time schools parks, restaurants, bars, cinemas, non-essential shops and services were closed in Ireland for 120 days from March 12th (Hale et al., 2021). In addition to school and business closures, people were expected to isolate in their homes and were permitted to exercise within a two-kilometre radius of their houses, in May 2020 this was extended to five kilometres. With the restrictions in place at this time, adolescents’ interaction with others outside of their family was dramatically reduced. Previous research combined with Bronfenbrenner’s (1979) Ecological Systems Theory will help inform the selection of factors which will be controlled for across levels. Based on the previous evidence, we hypothesize the following: (1) COVID-19 anxiety (comprised of Disease Anxiety and Consequence Anxiety) will be a significant predictor of psychological distress, even when controlling for other influencing factors; (2) Microsystem level factors (e.g., parent and peer) will moderate the relationship between COVID-19 anxiety and psychological distress.

## Methods

### Design

The results reported in this paper form part of the larger CoSPACE (COVID-19: Supporting Parents, Adolescents and Children during Epidemics) Ireland study, which is a longitudinal, online survey designed to track the mental health of parents and their children (aged 4–18 years old) throughout the COVID-19 pandemic in the Republic of Ireland. This paper focuses exclusively on cross-sectional data collected during the early stages of the COVID-19 pandemic, from both parents reporting on the mental health of their school-aged adolescent children (aged 11–18 years old) and the adolescents themselves.

### Procedure

Convenience sampling was used to recruit participants. This was achieved through a variety of means, including (social) media, targeted online advertising and distribution through partner organisations, networks and charities. The online survey was conducted via Qualtrics Online Software. Ethical approval was received from the University of Limerick’s ethics committee (ref: 2020_04_22_EHS). The survey was divided in two sections, the first to be completed by the parents and the second to be completed by the adolescent. Informed consent was obtained from both the parents and adolescents prior to their participation.

### Participants

A total of 314 adolescents and their parents completed the survey between April and June 2020. While adolescents’ self-report measures are the focus in this study, a selection of parent-reported measures have been included as covariates in the analysis.

Mean age was 14.05 years (Range 11–18 years). Males comprised the largest portion at 51% (*n* = 160), while females accounted for 48.7% (*n* = 153), which is largely in line with the national average. One respondent self-identified their gender as ‘not sure/questioning’ (0.3%). Most respondents were White Irish, at 91.7% (*n* = 288) (national average is 82.2%). Non-Irish White made up 3.8% (*n* = 12), Non-Chinese Asian made up 1% (*n* = 3), 2.9% comprised other including mixed background (*n* = 9), while the remaining responses were African (*n* = 1), and Chinese (*n* = 1), at 0.3% each. Most respondents’ (72.3%) annual household income was greater than €34,000 (*n* = 227), while 17.2% had an annual household income of less than €34,000 (*n* = 54). 9.6% of respondents did not wish to disclose their household income (*n* = 30), while the remaining 1% were missing responses (*n* = 3) (national average household income 2019 was €43,500). A majority (86.9%; *n* = 273) of respondents did not have an SEN. 13.1% (*n* = 41) were reported to have an SEN, which is significantly higher than the national average of 3.3%.

### Measures

#### Kessler psychological distress scale (K6)

Psychological distress was assessed using the Kessler Psychological Distress Scale (K6; Kessler et al., 2003). The scale consisted of six questions, where adolescents reported how often they had been feeling nervous, hopeless etc. during the past week. Responses were rated on a 5-point Likert scale and scored from 0 ‘none of the time’ to 4 ‘all of the time’. Scores were summed to obtain total scores with a possible range of 0 to 24, with higher scores indicating higher levels of psychological distress. In the current study, Cronbach’s α for the K6 scale was found to be 0.85, which indicates good internal consistency. The K6 scale has been demonstrated to be valid and reliable in an epidemiological sample of youth (Ferro, 2019). For the purpose of descriptive statistics, we report on risk for serious mental illness using the clinical cut-off score of >13, in line with (Umucu et al., 2022). However, the continuous score was used in the main analyses.

#### Pandemic anxiety scale

COVID-19 anxiety was assessed using the Pandemic Anxiety Scale (PAS), developed by McElroy et al. (2020). The 7-item PAS asked participants to rate their responses to seven statements, which assessed how they were feeling during the COVID-19 outbreak, on a 5-point Likert scale ranging from ‘strongly disagree’ (0) to ‘strongly agree’ (4). The PAS captures two forms of COVID-19 anxiety which were divided into two subscales: Disease Anxiety [measures anxiety surrounding the COVID-19 disease itself, four items, and Consequence Anxiety (measures anxiety surrounding the consequences of the COVID-19 pandemic and the resulting lockdowns, three items)]. Disease Anxiety items were “*I am worried I will catch COVID-19*,” ‘*I am worried that my friends and family will catch COVID-19*’, ‘*I am afraid to leave the house right now*’ and ‘*I am worried I might transmit the infection to someone else*’. Consequence Anxiety items were “*I am worried about missing schoolwork*,” ‘*I am worried about the amount of money we have coming in*’ and ‘*I am worried about the long-term impact which will have on my job prospects and the economy.*’ Total scores ranged from 0 to 28, with higher scores indicating higher levels of COVID-19 anxiety. Summed scores for Disease Anxiety and Consequence Anxiety were also calculated, with Disease Anxiety score potentially ranging from 0 to 16 and Consequence Anxiety score potentially ranging from 0 to 12. Although the PAS is a new scale, it has been found to be both reliable and valid, and suitable for use with adults and adolescents in large-scale survey studies. The measure was initially validated in a sample recruited in the first 6 weeks of Covid-19 lockdown, parents (*N* = 4,793) and adolescents (*N* = 698) in a UK population and demonstrated to be a reliable measure of two distinct types of anxieties arising due to the COVID-19 pandemic (disease anxiety and consequence anxiety). In the current study, the total COVID-19 anxiety scale had a Cronbach’s *α* = 0.77, which indicates acceptable internal consistency. For the Disease Anxiety subscale Cronbach’s *α* = 0.81, indicating good internal consistency. For the Consequence Anxiety subscale, Cronbach’s *α* = 0.66, which indicates questionable internal consistency, although above 0.6 is reported to be generally acceptable (Ursachi et al., 2015). Furthermore, McElroy et al. (2020) noted that the Cronbach’s Alpha can be unduly low in this instance as a result of the low number of items in this sub scale.

#### Covariates

##### Individual level covariates

Variables controlled for at the individual level included socio-demographic variables (i.e., Age, Gender, Ethnicity, SES) and a variable regarding the presence or absence of an SEN. Age and Gender were self-reported by the adolescents and Ethnicity, SES and SEN were reported by parents/caregivers on their adolescent children. Ethnicity was measured using a multiple-choice response where parents selected from options: White Irish, Irish Traveller, any other White background, African, any other Black background, Chinese, any other Asian background, other (including mixed background), and prefer not to say. Those that selected ‘other (including mixed background)’ were given the opportunity to provide more detail in an open text box. Given that 91.7% of the sample identified as White Irish, ethnicity was recoded into a binary variable reflecting White Irish and everyone else. SES was operationalized as a measure of total gross household income, with scaled response options ranging from < €18,000 to > €136,000 per year as follows: 1 = Under €18,000 per year (€350 per week), 2 = €18,000 to €34,000 per year (€350–€653 per week), 3 = €34,001 to €68,000 per year (€653–€1,307 per week), 4 = €68,001 to €102,000 per year (€1,307–€1961 per week), 5 = €102,001 to €136,000 per year (€1961–€2,615 per week), 6 = More than €136,001 per year (€2,615 per week) and 7 = Prefer not to say. Finally, SEN was assessed by the question “Does your child have any special educational needs?,” with the response options of yes or no.

##### Microsystem level covariates

Variables controlled for at the microsystem level included Peer Support, School Support and Parent Child Closeness. Peer Support and School Support were reported by parents and Parent Child Closeness was reported by the adolescents themselves. Peer Support was assessed by the degree to which parents agreed with the statement “*My child has at least one friend that they can turn to for support*” and School Support was measured by the degree to which parents agreed with the statement “*My child would still be able to turn to an adult at school for support if they needed to*.” Both variables were measured on single-item scales and scored on a four-point Likert scale (‘not at all,’ ‘a bit,’ ‘a lot’ and ‘completely’). Similar single-item scales have been demonstrated to be reliable measures of support in other population studies (Slavin et al., 2020; Gallagher et al., 2022). Parent proxy measures have also been found to be robust in other studies with youth (Erhart et al., 2009) although there are mixed findings on the use of such measures (Jokovic et al., 2004). Parent Child Closeness was measured though the question “*Overall, how close would you say you are to your parent(s)/caregiver(s)?*,” and again responses were scored on a four-point Likert scale (‘not very close,’ ‘fairly close,’ ‘very close’ and ‘extremely close’). This single item question was adapted from the Millennium Cohort Study Age 14 Sweep (Study, 2016 MCS Sweep 6, 2016).

### Statistical analyses

All statistical analyses were conducted using IBM SPSS Statistics Version 26.0. Frequencies, Means and Standard Deviations were firstly calculated to obtain descriptive information on the full sample of adolescents. Bivariate Pearson and Point-Biserial correlation analyses were then conducted to establish associations between PAS score (total and subscales) and K6 score as well as between all covariates and K6 score. Independent samples *t*-tests were carried out for binary categorical variables that were found to have a statistically significant relationship with K6 in the correlation matrix to further explore these associations. A hierarchical multiple regression analysis was employed to determine the unique relationship between COVID-19 anxiety (in the form of its two sub-constructs, disease anxiety and consequence anxiety) and adolescents’ psychological distress, with significant covariates identified in the correlation analysis inputted in the regression model The regression model was constructed as follows: first, the covariates situated at the individual level of the ecological environment were inputted, i.e., Age, Gender, SEN and Ethnicity; second, the covariates located within the microsystem level were inputted, i.e., Peer Support, School Support, Parent Child Closeness; t finally, Consequence Anxiety and Disease Anxiety were inputted. The dependent variable was K6 score.

Lastly, two simple moderation analyses were conducted using PROCESS macro version 3.5 for SPSS, to assess whether the microsystem level factors that were found to be significant predictors in the regression model (Peer Support, and Parent Child Closeness), moderated the relationship between Consequence Anxiety (which was identified as a significant predictor of K6 score when controlling for Disease Anxiety in the regression model) and K6 score. Significant covariates identified in the regression model, as well as Disease Anxiety, were controlled for in the moderation analyses. Changes in degrees of freedom reflect missing data.

## Results

### Descriptive statistics

Participants’ mean K6 score was 5.27 (*SD* = 4.99), with 11% of participants at high risk for serious mental illness (K6 score ≥ 13). The mean PAS score for participants was 16.72 (*SD* = 6.37) and the subscale means for PAS were 7.76 (*SD* = 3.68) for Disease Anxiety and 4.77 (*SD* = 3.04) for Consequence Anxiety. See Table 1 for Means and SDs for all other continuous variables included in the regression analyses.

**Table 1:** Means and standard deviations for included continuous variables (*N* = 314).

Variables	Mean ± SD	Range
Age (years)	14.05/2.7	11–18 years
Peer support	3.15/0.938	1–4
School support	2.81/1.03	1–4
Parent child closeness	3.24/0.799	1–4
Pandemic anxiety scale	16.72/6.37	0–28
PAS disease anxiety	7.76/3.68	0–16
PAS consequence anxiety	4.77/3.04	0–12
K6	5.27/4.99	0–24

A series of correlation analyses were run to test the association between K6 score and scores on all other possible predictor variables. When examining the associations between predictor variables and K6 score, zero order correlations were used and are presented in the correlation matrix table below (see Table 2).

**Table 2:** Correlation matrix of psychological distress and predictor variables.

	K6	Child age	Gender	Ethnicity	Income	Child SEN	Peer support	School support	Parent child closeness	PAS total score	PAS disease anxiety	PAS consequence anxiety
K6	–											
Child age	0.183**	–										
Gender	0.142*	0.093	–									
Ethnicity	0.203**	−0.007	0.099	–								
Income	−0.053	0.053	−0.009	−0.070	–							
Child SEN	−0.199***	0.142*	0.145*	−0.089	0.117*	–						
Peer support	−0.161**	0.218***	0.109	−0.121*	0.033	0.242***	–					
School support	−0.176**	0.053	−0.041	−0.237***	0.001	0.058	0.340***	-				
Parent child closeness	−0.260***	−0.299***	−0.009	−0.173**	−0.056	0.005	−0.016	0.233***	–			
PAS total score	0.403***	0.207***	0.097	0.118	−0.046	−0.081	−0.068	−0.204***	−0.068	–		
PAS disease anxiety	0.282***	0.070	0.035	0.094	0.001	−0.129*	−0.058	−0.116	0.007	0.855***	–	
PAS consequence anxiety	0.433***	0.350***	0.171**	0.103	−0.071	−0.019	−0.043	−0.204***	−0.146**	0.786***	0.393***	–

*p < 0.05, **p < 0.01, ***p < 0.001. SEN: 0 = Yes, 1 = No; Gender: 0 = Male, 1 = Female; Ethnicity: 0 = White Irish, 1 = Everyone else.

### Independent samples *t*-tests

Correlation analyses revealed that three categorical variables were statistically significantly correlated with K6 score, namely, whether the adolescent has an SEN, adolescent gender and ethnicity. A series of independent samples *t*-tests revealed there was a significant difference in K6 score between adolescents with a SEN (*M* = 7.81, *SD* = 5.33), and adolescents without an SEN (*M* = 4.89, *SD* = 4.83), *t*(270) =3.333, *p* < 0.001. A second independent *t*-test found that there was also a statistically significant difference in K6 score between adolescent males (*M* = 4.54, *SD* = 3.96), and adolescent females (*M* = 5.94, *SD* = 5.72), *t*(269) = −2.346, *p* = 0.020. A third independent t-test found that there was also a statistically significant difference in K6 score between adolescents who were identified as White Irish (*M* = 4.97, *SD* = 4.68) and everyone else (*M* = 8.68, *SD* = 6.92), *t*(269) = −2.466, *p* = 0.022.

### Hierarchical multiple regression analyses

A hierarchical multiple regression analysis was conducted to examine whether Consequence Anxiety and Disease Anxiety significantly predicts psychological distress (K6 score) in adolescents, while controlling for several influencing factors across individual (i.e., Age, Gender, SEN, Ethnicity) and micro-system levels (i.e., Peer Support, School Support, Parent Child Closeness).

The first step of the model, which included individual level covariates (e.g., Age, Gender, SEN, and Ethnicity) was significant, *F*(4,255) = 9.344, *p* < 0.001, adjusted *R^2^* = 0.114. As can be seen in Table 3, Age, Gender, SEN and Ethnicity were significant predictors of K6 score with this first step of the model explaining 11.4% of the variance in K6 score.

**Table 3:** Summary of hierarchical multiple regression analysis for variables predicting psychological distress.

***N* = 260**	**Step 1**					**Step 2**					**Step 3**				
	***B***	***β***	***R*** ^**2**^	**Adjusted *R*** ^**2**^	***R*** ^**2**^ **change**	***B***	***β***	***R*** ^**2**^	**Adjusted *R*** ^**2**^	***R*** ^**2**^ **change**	***B***	***β***	***R*** ^**2**^	**Adjusted *R*** ^**2**^	***R*** ^**2**^ **change**
**Constant**	6.241***	–	0.128***	0.114***	0.128***	10.456***	–	0.185***	0.162***	0.057***	6.945***	–	0.293***	0.267***	0.108***
**Age**	0.386**	0.179		–		0.312*	0.145		–		0.057	0.027			–
**Gender (ref = male)**	1.204*	0.122		–		1.355*	0.137		–		0.955	0.097			–
**SEN (ref = having a SEN)**	−3.477***	−0.228		–		−2.827**	−0.185		–		−2.559**	−0.168			–
**Ethnicity (ref = White Irish)**	3.476***	0.192		–		2.389*	0.132		–		2.029*	0.112			–
**Peer Support**	–	–		–		−0.710*	−0.134		–		−0.628	−0.118			–
**School support**	–	–		–		–0.216	−0.045		–		0.143	0.030			–
**Parent child closeness**	–	–		–		−1.170**	−0.190		–		−1.211***	−0.196			–
**Disease anxiety**	–	–		–		–	–		–		0.146	0.108			–
**Consequence anxiety**	–	–		–		–	–		–		0.508***	0.306			–

Model = “Enter” method in SPSS Statistics; B = unstandardized regression coefficient; β, standardized coefficient; *R*^2^, *R* square. SEN: 0 = Yes, 1 = No; Gender: 0 = Male, 1 = Female; Ethnicity: 0 = White Irish, 1 = Everyone else. *p < 0.05, **p < 0.01, ***p < 0.001.

The second step of the model including the microsystem level covariates (e.g., Peer Support, School Support and Parent Child Closeness) led to a significant change in the model; *F*(7,252) = 8.158, *p* < 0.001, adjusted *R^2^* = 0.162, *R^2^* change *=* 0.057. As displayed in Table 3, Peer Support and Parent Child Closeness together with the step 1 variables account for 16.2% of the variance in K6 score.

The final step of the model including the addition of Consequence Anxiety and Disease Anxiety was associated with a significant change to the model (*p* < 0.001) and the overall model was significant, *F*(9,250) = 11.507, *p* < 0.001, adjusted *R^2^* = 0.267, *R^2^* change = 0.108. As shown in Table 3, Consequence Anxiety was a significant, positive predictor of K6 score, while controlling for covariates and Disease Anxiety. Disease Anxiety was a non-significant predictor in K6 score. The final model for Consequence Anxiety and Disease Anxiety, together with the step 1 and 2 variables accounted for 26.7% of the variance in K6 score.

### Moderation analyses

Two simple moderation analyses were conducted to determine if the significant microlevel factors that were significantly associated with psychological distress (Peer Support and Parent Child Closeness), moderated the relationship between Consequence Anxiety and K6 score.

**Figure 1:**
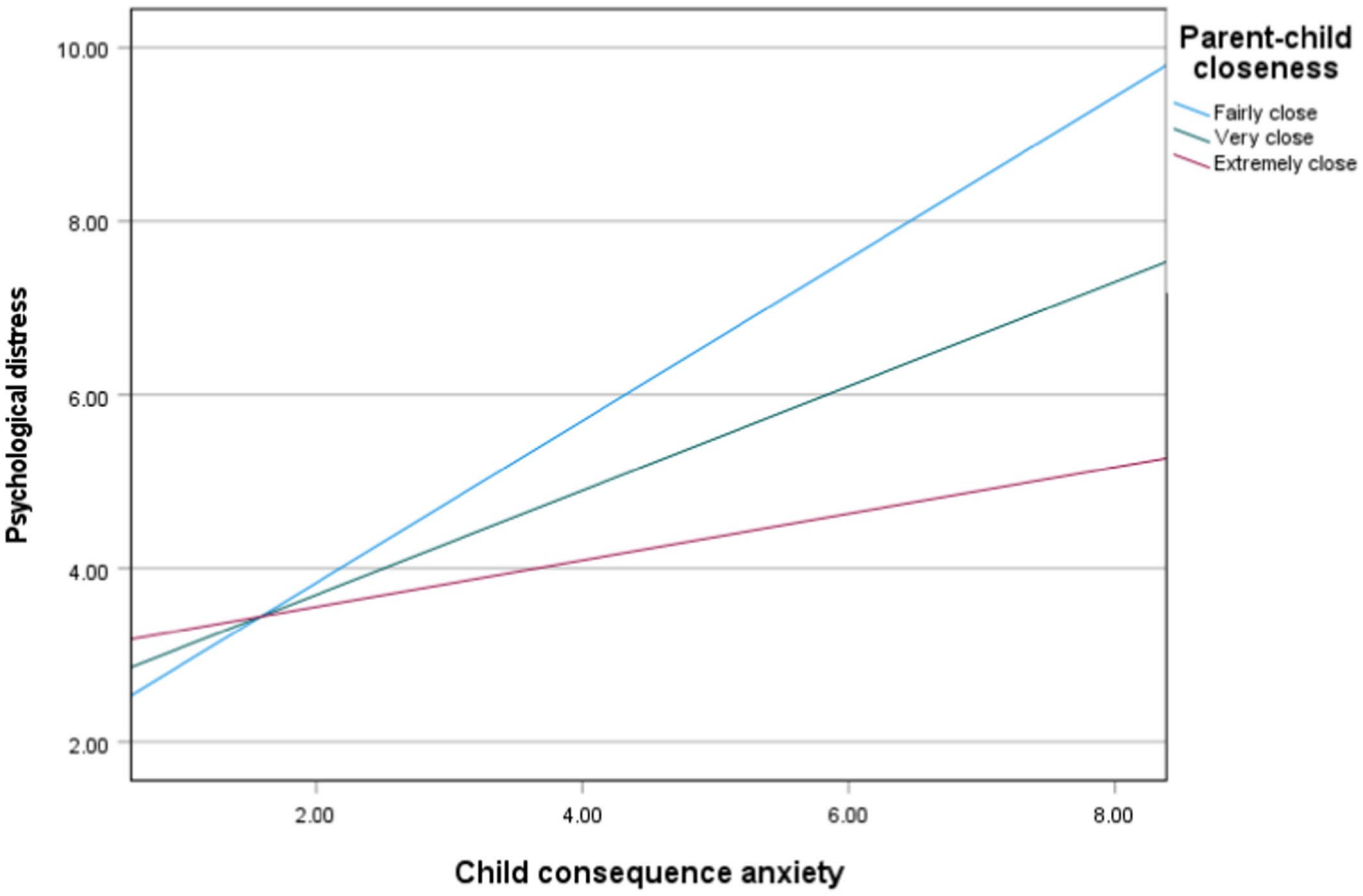
The moderation of parent–child closeness on the relationship between child consequence anxiety and psychological distress. Age, gender, SEN, peer support and disease anxiety are included as covarieties. Reference category of the moderator = 1 -Not very close.

For the first moderation, covariates in the model include age, gender, SEN, ethnicity, peer support and disease anxiety. The overall model was significant; *F*(9, 250) = 12.99, *p* < 0.001, *R^2^* = 0.32. The Parent–Child-Closeness*Consequence Anxiety interaction was significant, with greater Parent–Child Closeness significantly reducing the strength of the positive association between Consequence Anxiety and Psychological Distress; *B* = –0.33, *t*(250) = −3.12, *p* = 0.002. Figure 1. depicts the Parent–Child-Closeness*Consequence Anxiety interaction. Finally, the main effect of Consequence Anxiety had a significant positive association with K6 score; *B* = 1.60, *t*(250) = 4.35, *p* < 0.001. The main effect of Parent Child Closeness was not significantly associated with K6 scores; *b* = 0.53, *t*(250) = 0.820, *p* = 0.413.

As the data presented are cross-sectional, the alternative moderation model was also checked (i.e., would Parent Child Closeness moderate the association between K6 scores as the predictor and Consequence Anxiety as the outcome). Covariates include age, gender, SEN, ethnicity, peer support and disease anxiety. The overall model was significant; *F*(9, 250) = 14.37, *p* < 0.001, *R^2^* = 0.34, but the interaction effect [*B* = –0.01, *t*(260) = −0.36, *p* = 0.72] and main effects for K6 score [*B* = 0.21, *t*(260) = 1.90, *p* = 0.06] and Parent Child Closeness [*B* = 0.05, *t*(260) = 0.16, *p* = 0.88] were non-significant, indicating that the original model best predicts adolescent psychological distress and fits within our theoretical framework.

For the second moderation, covariates in the model include age, gender, SEN, ethnicity, parent–child closeness and disease anxiety. The overall model was significant; *F*(9, 250) = 11.47, *p* < 0.001, *R^2^* = 0.29. The Peer Support*Consequence Anxiety interaction was non-significant; *B* = 01, *t*(250) = 0.06, *p* = 0.95. Furthermore, main effects were non-significant for Consequence Anxiety; *B* = 0.48, *t*(250) = 1.49, *p* = 0.14 and Peer Support; *B* = -0.61, *t*(250) = −1.13, *p* = 0.26.

## Discussion

This study examined the unique relationship between COVID-19 anxiety and psychological distress in a sample of Irish adolescents during the early stages of the COVID-19 pandemic. As hypothesized, higher levels of COVID-19 anxiety predicted higher levels of psychological distress, whilst controlling for a variety of covariates situated at multiple levels of Bronfenbrenner’s (1979) ecological framework.

Specifically, consequence anxiety (when entered in the regression model alongside disease anxiety) accounted exclusively for 10.8% of the variance in adolescents’ psychological distress, and the overall hierarchical multiple regression model accounted for 26.7% of the variance in adolescents’ psychological distress, when controlling for age, gender, SEN, ethnicity, peer support, school support and parent child closeness. Disease Anxiety did not significantly predict distress when consequence when simultaneously entered into the regression model with Consequence Anxiety, suggesting that the anxiety related to the consequences of contracting COVID-19 was the key predictor of distress. This important finding is in line with Magson et al.’s (2021) discovery that adolescents were more concerned about the government restrictions implemented to prevent the COVID-19 viruses’ spread than they were about the virus itself. Indeed, the comparatively lower levels of anxiety/concern surrounding the COVID-19 virus may be at least partially explained by the extremely low adolescent COVID-19 mortality rate and the low chances of adolescents becoming seriously ill from COVID-19 (Bhopal et al., 2021), unlike older adults who are at greater risk of having underlying health conditions that can be exasperated by COVID-19 (Hyland et al., 2020). Meanwhile, the higher levels of anxiety/concern regarding the consequences of the COVID-19 pandemic may be understood by considering the severe impact of the public safety measures which were implemented to contain the virus. Isolation, lack of access to schooling and limited contact with peers, could have had serious consequences on young people during such a critical developmental period (de Figueiredo et al., 2021). Theoretically our findings are consistent with an ecological systems approach to anxiety in youth, which emphasises bi-directional influences ranging from proximal (i.e., child age, gender, SEN) to distal influences (i.e., parent relationships) (Mian et al., 2011). Youth may be more concerned about the consequences of COVID-19 if they are predisposed to worry about associated restrictions (de Figueiredo et al., 2021), have concern about transmitting the virus and fear future uncertainty (Saurabh and Ranjan, 2020). This can be compounded when family relationships are poor or worsen (Kılınçel et al., 2021).

Factors at the individual level, such as Age, Ethnicity and SEN were also found to determine adolescents’ levels of psychological distress during the COVID-19 pandemic in this study. Existing research supports the finding that these individual factors, e.g., age (i.e., being older) (Zhou et al., 2020), belonging to an ethnic minority group (i.e., Darmody et al., 2020) and presence of an SEN (Sibley et al., 2021), are all risk factors for psychological distress of adolescents.

Within the microsystem, family relationship was found to be an important factor for reducing psychological distress during the COVID-19 pandemic. In this study, Parent Child Closeness was a significant predictor of psychological distress, with the presence of a ‘very’ or ‘extremely close’ relationship predicting lower levels of psychological distress. Due to public safety advice advising citizens to stay at home as much as possible during periods of lockdown in the COVID-19 pandemic, adolescents’ social relations have been mainly restricted to close family members (Fegert et al., 2020). While increased time with family members may have profound negative impacts on mental health in some circumstances (e.g., increased exposure to domestic violence) (Fegert et al., 2020) the presence of positive familial relations during the COVID-19 pandemic, can also positively impact on mental health outcomes. Strengthening familial bonds (e.g., increased sense of closeness between parents and adolescents), may act as a powerful protective factor against adolescents’ psychological distress during the pandemic. This was not only evident in this study but has been supported by empirical research which found that closeness with parents during adolescence, can act as buffer against adverse mental health outcomes (Ge et al., 2009; Oberle et al., 2011; Wang et al., 2020; Hartas, 2021; Nolan and Smyth, 2021). Peer Support was also found to be a significant predictor of psychological distress, with the presence of Peer Support (i.e., at least one friend the adolescent could turn to) predicting less psychological distress. This finding is consistent with previously conducted empirical research, e.g., Magson et al.’s (2021) reporting on social connectedness as a protective factor against adolescents’ poor mental health during the COVID-19 pandemic. Erikson’s model of psychosocial development suggests that social relationships are of great importance during the adolescent years as hormonal changes make adolescents more highly attuned to social status, peer groups and relationships (Imran et al., 2020). Conversely, adolescents may find public safety measures during the COVID-19 pandemic, such as social distancing, particularly difficult, as their needs for social connection and acceptance by peers can go unmet (Andrews et al., 2020; Fegert et al., 2020). A study by McMahon et al. (2020) which reports on the national longitudinal Growing Up in Ireland (GUI) survey data found that while stressful life events can negatively impact on the psychological wellbeing of adolescents, parent and peer relationships can mediate this association, particularly for adolescent girls.

A series of moderation analyses were conducted to determine whether microsystem level factors moderated the relationship between Consequence Anxiety and psychological distress, while controlling for individual-level factors and disease anxiety. Whilst peer support was not a significant moderator in the relationship between consequence anxiety and psychological distress, parent child closeness was found to moderate the relationship between consequence anxiety and psychological distress, with the influence of Consequence Anxiety on Psychological Distress lesser for participants with closer parent–child relationships. This indicates that closer child/parent relationships attenuate the effect of anxiety about the consequences of COVID-19 on psychological distress. As such child/parental closeness is an important protective and modifiable factor with respect to adolescents’ worries and distress relating to the effect of COVID-19. Our finding also aligns with prior research (Moretti and Peled, 2004; Ackard et al., 2006), which has highlighted that the adolescent-parent attachment bond is critical in supporting adolescents during difficult periods.

Therefore, strategies to reduce psychological distress during COVID-19 should focus directly on reducing pandemic anxiety, in particular consequence anxiety, but also on family supports that promote greater parent child closeness and can buffer against the negative effects of consequence anxiety.

### Implications

The findings reported in this paper have several practical implications which are of value to health professionals, practitioners and researchers for improving psychological distress outcomes during pandemics. Most importantly, after controlling for individual and microsystem level factors, COVID-19 anxiety was found to predict psychological distress in adolescents. Specifically higher consequence anxiety related to COVID-19 was associated with increased psychological distress. This suggests that messaging about the impact of COVID-19 or other pandemic-related events, particularly the consequences of such events, is a stressor for youth and should receive more attention as an important factor leading to psychological distress during pandemics.

For healthcare providers, measures of COVID-19 anxiety could be utilized to screen for adolescents who may be at risk of developing mental health issues. Similarly, understanding the individual and micro-level risk factors (e.g., age, gender SEN, poor family relationships) for adolescents can help identify those that require additional support. Tailored therapeutic interventions could target adolescents presenting with these risk factors, specifically those with high consequence anxiety, to mitigate the potential development of negative mental health outcomes for these vulnerable adolescents (Talevi et al., 2020).

Furthermore, findings around COVID-19 anxiety could be used to inform the design and implementation of therapeutic interventions by specifically addressing COVID-19 anxiety and decreasing adolescents’ levels of psychological distress. In line with Lee et al.’s (2020) recommendations, it might therefore be useful to offer internet-based Cognitive Behavioural Therapy (CBT) during pandemics to adolescents who display elevated levels of COVID-19 anxiety and to implement this therapy to address and reduce COVID-19 anxiety and levels of psychological distress. Indeed, our findings suggest that COVID-19 anxiety is an important predictor of adolescent psychological distress and as such directly intervening with COVID-19 anxiety will have benefits for adolescents’ mental health outcomes. Enhancing the parent child relationship was also highlighted as a possible avenue of intervention, with findings indicating that a positive child/parental relationship may buffer against psychological distress linked to consequence anxiety related to the pandemic. This aligns with Zhou (2020) who advocates for a multi-systemic approach that encompasses psychological support for adolescents and those they are influenced by (e.g., family, peers, teachers etc.). In future research, subsequent studies could employ a longitudinal design to investigate how COVID-19 anxiety, psychological distress and the predictive relationship between them changes over the course of the pandemic. Future research should also concern itself with the after-effects of COVID-19 anxiety in the post-COVID-19 world, and whether any long-term adverse consequences of the pandemic on the mental health of adolescents can be identified. This line of research is perhaps the most critical in determining whether and to what extent the COVID-19 pandemic and its resulting anxiety has affected adolescent development and whether any adverse mental health effects experienced during the pandemic by adolescents, who are undergoing a crucial developmental period, result in psychopathological conditions in early and later adulthood (de Figueiredo et al., 2021).

### Strengths and limitations

The study is, as far as we are aware, the first to examine whether COVID-19 anxiety is uniquely related to the mental health of adolescents during the COVID-19 pandemic. The identification of COVID-19 anxiety, particularly consequence anxiety, as a key risk factor for increased psychological distress in adolescents during the pandemic has therefore filled a major gap in the literature and followed an avenue of research that has not previously been explored. The study has also added to the limited literature on the mental health impact of the COVID-19 pandemic and has thus shed light on the mental health costs of the pandemic for youth.

Limitations of the study should also be considered. First, the study employed a cross-sectional design and was conducted during the early phases of the COVID-19 pandemic, meaning that the findings may not be applicable to later stages of the pandemic (Xiong et al., 2020). The cross-sectional nature of the study is limited in its ability to account for the predictive relationship between COVID-19 anxiety and adolescents’ psychological distress throughout the pandemic. Furthermore, the cause-effect relationship between COVID-19 anxiety and psychological distress cannot be established. Second, caution must also be taken in assuming generalizability of these findings outside of the Irish context as statistics from the World Health Organization (2021) have shown that parts of the world have been differentially impacted by the COVID-19 pandemic. For example in June 2020 deaths from COVID-19 ranged from as low as 4 deaths per million in Australia to as high as 829 deaths per million in Belgium (Balmford et al., 2020). Similarly containment restrictions ranged from ‘no restrictions at all’ to ‘maximum containment’ at varying times through the initial wave (Cascini et al., 2022). Third, participants were recruited through convenience sampling and the study is therefore not representative of Irish adolescents at a national level. The self-selecting nature of the survey may also have resulted in a sampling bias, wherein some groups of adolescents were over-represented in the survey, while others went under-represented. In particular, the online format of the survey may have led to sampling bias because only parents and adolescents with access to a computer or smartphone would have been able to complete the survey, with this ‘digital divide’ possibly leading to under-representation of socio-economically disadvantaged families (Darmody et al., 2020). Indeed, most participants reported their gross household income to be >€34,000 *per annum*, and it is possible that this exclusion of very low-income families may have resulted in an under-estimation of adolescents’ COVID-19 anxiety and psychological distress levels. The sample was also predominantly White Irish (91.7%) limiting generalisability to ethnic minorities. A further limitation is that single-item scales were used to assess microsystem factors. Additionally, some of these scales (Peer Support and School Support) were completed by parents (not adolescents themselves). There are mixed findings on the use of parent proxy measures (Jokovic et al., 2004; Erhart et al., 2009), which limits the reliability of these measures and can potentially lead to discrepancies in reporting (Kim et al., 2020). Finally, Cronbach’s α for the Consequence Anxiety subscale of the PAS was <0.70, which indicates questionable internal consistency. Although prudent interpretation of the findings in relation to consequence anxiety is warranted, McElroy et al. (2020) does suggest that the low alpha value can be explained by the low number of items of the scale.

## Conclusion

This study indicated that COVID-19 anxiety is a predictor of psychological distress during the COVID-19 pandemic in a sample of Irish adolescents, particularly consequence anxiety. The identification of consequence anxiety as a key risk factor for adolescents’ psychological distress during the pandemic has important practical implications. COVID-19 anxiety may serve as both an important indicator for identifying adolescents at-risk of developing psychological distress during pandemics and provide an area to focus on when developing strategies and interventions to mitigate these negative outcomes. The identification of predictors of psychological distress at both the individual level and microsystem level of Bronfenbrenner’s (1979) ecological environment also suggests that a multisystemic approach, particularly targeting the parent child relationship, is best suited to reduce the negative mental health impacts of the pandemic on adolescents. Research, policy and practice should consider these findings to help strengthen future studies, risk identification and therapeutic intervention development.

## Data availability statement

The raw data supporting the conclusions of this article will be made available by the authors, without undue reservation.

## Ethics statement

The studies involving humans were approved by Ethics Research Committee Faculty of Education and Health Sciences University of Limerick. The studies were conducted in accordance with the local legislation and institutional requirements. Written informed consent for participation in this study was provided by the participants’ legal guardians/next of kin.

## Author contributions

JM, EG, SH, CO’C, MR, and EW contributed to study design and data collection. JM, AD, and KD contributed to data interpretation. All authors contributed to the write-up of the article. EG and EW contributed to data anlaysis.
